# CRRTnet: a prospective, multi-national, observational study of continuous renal replacement therapy practices

**DOI:** 10.1186/s12882-017-0650-2

**Published:** 2017-07-06

**Authors:** Michael Heung, Sean M. Bagshaw, Andrew A. House, Luis A. Juncos, Robin Piazza, Stuart L. Goldstein

**Affiliations:** 1 Division of Nephrology, Department of Medicine, University of Michigan, Ann Arbor, MI USA; 2 Department of Critical Care Medicine, Faculty of Medicine and Dentistry, University of Alberta, Edmonton, AB Canada; 3 Division of Nephrology, Department of Medicine, University Hospital London Health Sciences Centre, London, ONT Canada; 4 Department of Medicine/Nephrology, and Department of Physiology and Biophysics, University of Mississippi Medical Center, Jackson, MS USA; 5 Watermark Research Partners, Inc., Indianapolis, IN USA; 6 Center for Acute Care Nephrology, Cincinnati Children’s Hospital Medical Center, Cincinnati, OH USA; 7 1500 E. Medical Center Drive, SPC 5364, Ann Arbor, MI 48109-5364 USA

**Keywords:** Acute kidney injury, Critical care, Continuous renal replacement therapy, Quality

## Abstract

**Background:**

Continuous renal replacement therapy (CRRT) is the recommended modality of dialysis for critically ill patients with hemodynamic instability. Yet there remains significant variability in how CRRT is prescribed and delivered, and limited evidence-basis to guide practice.

**Methods:**

This is a prospective, multi-center observational study of patients undergoing CRRT. Initial enrollment phase will occur at 4 academic medical centers in North America over 5 years, with a target enrollment of 2000 patients. All adult patients (18–89 years of age) receiving CRRT will be eligible for inclusion; patients who undergo CRRT for less than 24 h will be excluded from analysis. Data collection will include patient characteristics at baseline and at time of CRRT initiation; details of CRRT prescription and delivery, including machine-generated treatment data; and patient outcomes.

**Discussion:**

The goal of this study is to establish a large comprehensive registry of critically ill adults receiving CRRT. Specific aims include describing variations in CRRT prescription and delivery across quality domains; validating quality measures for CRRT care by correlating processes and outcomes; and establishing a large registry for use in quality improvement and benchmarking efforts. For initial analyses, some particular areas of interest are anticoagulation protocols; approach to fluid overload; CRRT-related workload; and patient safety.

**Trial registration:**

Registered on ClinicalTrials.gov 1/10/2014: NCT02034448.

## Background

Acute kidney injury (AKI) occurs commonly in critically ill patients, complicating more than half of intensive care unit (ICU) admissions [1, 2]. About 5–6% of ICU patients will receive renal replacement therapy (RRT), and hospital mortality rates in this population approach or exceed 50% [3–6]. Despite the major clinical significance of this syndrome, there remains no proven therapy to reverse or attenuate established AKI, and care is primarily supportive, including RRT in severe cases.

For ICU patients, particularly those with hemodynamic instability, continuous renal replacement therapy (CRRT) has emerged as the dialysis modality of choice in high-income nations. Since the original descriptions of continuous arteriovenous hemofiltration over 30 years ago, [7] CRRT has undergone significant evolution with several improvements allowing more widespread and routine use. Advancements include peristaltic pump driven venovenous circulation, sophisticated hardware and software engineering for precise application of the prescribed treatment and enhanced safety and alarms, improved biocompatibility and other membrane characteristics, regional anticoagulation protocols and enhancements in user interface. Indeed, for hemodynamically unstable patients the 2012 Kidney Disease: Improving Global Outcomes (KDIGO) AKI guidelines suggest using CRRT over intermittent dialysis options [8]. Compared to standard intermittent hemodialysis (IHD), CRRT offers advantages of improved hemodynamic stability, better overall solute clearance, and better fluid balance [8–10]. Observational studies also suggest higher rates of renal recovery among patients initially treated with CRRT compared to conventional IHD, although this benefit has not been observed in randomized trials [11].

While the clinical role of CRRT has become well-established, there exists tremendous variability in how CRRT is prescribed and delivered [12–14]. The KDIGO AKI guidelines provide some recommendations, stating that the target delivered CRRT effluent dose should be 20–25 mL/kg/h, and also suggesting that regional citrate should be the first-line form of anticoagulation [8]. However, specific recommendations are lacking for a number of other important areas, such as CRRT modality, details of anticoagulation protocols, electrolyte supplementation practices, or approach to fluid balance. This reflects the lack of strong evidence upon which to base guidelines, as most studies examining CRRT practices have been relatively small and/or single-center. In addition, despite the high-risk nature of the patient population, very few studies have examined quality metrics and/or patient safety related to CRRT practices [15].

Here we describe the protocol for a prospective observational study focused on CRRT practices in critically ill adult patients across a network of medical centers in North America (CRRTnet). Our goal is to develop a large repository of data regarding CRRT practices and outcomes, allowing characterization of similarities and differences in standard CRRT practices across medical centers. The natural variability in practices between (and even within) centers will provide the ability to compare outcomes between different practice patterns and generate important preliminary data to inform future interventional trials. In addition, a long-term goal for CRRTnet is to establish benchmarks for best CRRT practice for centers both within and outside the study.

## Methods/design

### Study oversight and consent

This study was registered on ClinicalTrials.gov before initiation of recruitment (identifier NCT02034448). Each of the initial recruitment centers has received local institutional review board approval for CRRTnet with a waiver for informed consent. This waiver was granted due to the minimal risk nature of this observational study, as well as the recognition that this study would not be practical without the waiver. Specifically, since no interventions are involved, patient recruitment into the database may occur on a retrospective basis and it would be inefficient and possibly emotionally distressing to contact patients or their families for consent after the fact. Furthermore, it is important to include all CRRT patients in order to provide an unbiased perspective of practice patterns and maximize validity of our findings.

Ongoing study oversight will be performed by the CRRTnet registry board, which is chaired by SLG and comprised of the site principal investigators for each of the recruitment centers.

### Design and setting

This is a prospective, multi-center observational study of critically ill adults undergoing CRRT. Initial recruitment is planned at 4 academic medical centers in North America with significant (>50 patients per year) volume CRRT use: University of Michigan Health System (Ann Arbor, MI), University of Alberta Hospital, University of Alberta (Edmonton, Alberta), University Hospital London Health Sciences Centre (London, Ontario), and University of Mississippi Medical Center (Jackson, MS).

### Patient population

All subjects in this study will be critically ill adult patients with AKI receiving CRRT as part of their standard of care. Inclusion criteria are age ≥ 18 years and ≤89 years. Exclusion criteria are patients who receive CRRT for less than 24 h, and those who are initiated on CRRT at an outside hospital prior to transfer in whom incomplete data are available. For patients who require multiple courses of CRRT during an admission, only the data from the first course will be considered. Patients with pre-existing end-stage renal disease on maintenance dialysis are also excluded.

### Data collection

Data will be collected encompassing a wide range of variables examining patient characteristics at enrollment, describing daily ICU course, capturing details of CRRT, and describing patient outcomes. Each participating medical center utilizes an electronic medical record which will serve as the primary source for data extraction.

The initial intake form includes demographic variables, and baseline patient characteristics at the time of CRRT initiation (Table 1). Fluid balance (in liters) between the time of ICU admission and CRRT initiation will be collected and used to calculate percent fluid overload using the following formula: [16, 17].

**Table 1:** Planned data collection at time of enrollment (CRRT initiation)

Category	Data Elements
Demographics	Date of birth/Age
	Gender
	Body mass index
	Hospital admission weight
Comorbidities	Components of Charlson Index
ICU Admission Data	ICU admission date
	Admission source (hospital ward, emergency department, outside hospital transfer)
	Primary condition
	Secondary condition
CRRT Initiation Data	Clinical indication for CRRT (all that apply)
	Time from ICU admission to CRRT initiation
	Cumulative fluid balance from ICU initiation to CRRT initiation
Laboratory Data	Serum creatinine at ICU admission
	Serum creatinine at CRRT initiation
	APACHE II score at ICU admission

*CRRT* continuous renal replacement theray, *ICU* intensive care unit

%fluid overload = [(total fluid intake (L) – total fluid output (L))/ICU admission weight (kg)] x 100%. Additional patient clinical data will be updated on a daily basis to reflect clinical management, severity of illness and laboratory findings (Table 2). All laboratory values will be recorded as available, and no laboratory testing will be requested as part of CRRTnet.

**Table 2:** Daily clinical data collection

Category	Data Elements
Severity of Illness	SOFA score components
	Need for mechanical ventilation
	Vasopressor requirements (drug and rate)
Fluid Balance	Type and volume of fluid intake
	Type and volume of recorded outputs
Nutrition	Enteral nutrition formula and volume
	Parenteral nutrition protein, calories and volume
Laboratory Data	Complete blood count: white blood cell count, hemoglobin, hematocrit, platelets
	Metabolic panel: sodium, potassium, chloride, bicarbonate, blood urea nitrogen, creatinine, phosphate, magnesium, albumin
	Coagulation parameters: INR, PTT, anti-Xa activity
	Blood gas: pH, PaO2, PaCO2, lactate, ionized calcium

For up to the first 7 days of a patient’s CRRT course, data regarding CRRT prescription and delivery will be recorded twice daily (Table 3). For days 8 thru 14, CRRT data will be captured once daily. Details of the CRRT prescription will be truncated at 14 days. Each of the initial recruitment centers performs CRRT utilizing the Prismaflex™ device (Baxter International Inc.) which has a datacard to which machine treatment data are automatically saved. These data include detailed information such as pressure measurements, fluid removal, delivered CRRT effluent dose, and all alarms, interruptions and interventions. Machine treatment data files will be downloaded and analyzed for each patient. Each site will de-identify the patient file using the de-identified patient ID assigned by the registry database in place of the patient identifier. The de-identified machine data files will be uploaded by the site into the CRRTnet Registry within each patient’s record. Data is parsed by the application and stored in the database into select data fields based on dates and time points for each day the patient is on CRRT. If the data files cannot be accessed/used, the site can manually fill in the data fields for the specified time points with data collected by the medical staff.

**Table 3:** Daily CRRT data collection

Category	Data Elements
Vascular Access	Dialysis catheter position
	Dialysis catheter type (acute or tunneled)
	Dialysis catheter manufacturer
	Dialysis catheter size
	Any need for catheter manipulation (e.g. repositioning, replacement)
CRRT Circuit	Any circuit interruptions/replacements and reasons
	Priming solution (new circuits)
CRRT-Related Anticoagulation	Anticoagulation type (heparin, citrate, none, other)
	Anticoagulation dose
	Anticoagulation metrics
CRRT Fluids	Type of solution
	Modality (dialysate, pre- and/or post-replacement) and rate
	Solution additives
CRRT Dose	Prescribed effluent dose
Machine Data Card	Delivered effluent dose
	Type, number and duration of machine alarms and interruptions
	Filter life

*CRRT* continuous renal replacement theray

Data entry will be performed by trained study staff at each site, and will be entered into an online system using a password-protected web-based research interface (Watermark EDGE, Watermark Research Partners, Inc.). Electronic data transmission will utilize secure socket layer (SSL) encryption technology to maintain compliance with privacy and data security. Data within the Watermark EDGE database is protected by Transparent Data Encryption (TDE) encrypted with the AES-265 Cipher. Limited Data Set (LDS) will be collected with regards to specific dates for the following: year of birth, admission dates, treatment dates, date of discharge or death. For patient data entry each patient will be de-identified via assigned unique codes and will not contain direct traceable identifiers. This registry complies with the following as set forth in 45 CFR Parts 160 and 164 (“HIPAA Privacy Rule”): “Covered Entity” (45 CFR § 160.103), “De-identified information” (45 CFR § 164.514), “Protected Health Information (“PHI”)” (45 CFR § 164.103) and “Limited Data Set (“LDS”)” (45 CFR 164.514(e)(2)). All sites will utilize the same electronic report forms, with the exception of measurement units customized to match each center’s customary laboratory reporting. For purposes of analysis all unit values entered by the center will also be converted and stored in a standardized unit for reporting in addition to the original entry.

### Interventions

CRRTnet is strictly an observational study and no clinical interventions will be performed. In addition, no biosample collection or storage will occur as part of CRRTnet.

### Operational definitions

1. Complete recovery: - Return of serum creatinine (SCr) to within 50% of baseline. Baseline SCr will be determined by the order of the following:

i. pre-hospital/outpatient SCr values
ii. lowest available in-hospital SCr (before or after) receiving CRRT
iii. back calculation for a SCr based on an eGFR 75 mL/min using the CKD-EPI equation

2. Partial recovery: - No longer receiving RRT but SCr has not returned to within 50% of baseline.
3. Non-recovery: - Remains dialysis dependent.

All serious adverse events related to CRRT will be recorded, with a specific focus on the following complications:

1. Major bleeding event related to heparin anticoagulation:

i. Drop in hemoglobin of ≥1.0 g/dL within a 24 h period, *and*
ii. Transfusion requirement of ≥2 units packed red blood cells

2. Development of heparin-induced thrombocytopenia:

i. Fall in platelets of at least 30% *and*
ii. Positive confirmatory test for heparin-induced thrombocytopenia (serotonin release assay)

3. Hypocalcemia event related to citrate anticoagulation:

i. Fall in systemic ionized calcium below 0.8 mmol/L, *and one of the following*
ii. Worsening hypotension/increasing pressor requirement, *or*
iii. Cardiac arrhythmia

All serious adverse events will be reviewed with each site’s principal investigator to confirm appropriateness for inclusion.

### Primary and secondary outcomes

The primary outcome will be patient survival at ICU discharge. Patient outcomes will also be recorded at hospital discharge. Additional outcomes include ICU and hospital length of stay, renal function recovery during hospitalization (defined above), and adverse events related to CRRT (defined above).

### Sample size

Due to the observational nature of CRRTnet, a formal sample size calculation was not performed. However, our target recruitment is 2000 patients, which would result in the largest and most detailed collection of CRRT patients to date.

### Statistical analysis

All descriptive data will be analyzed using standard statistical methods. CRRTnet will assess for potential associations between patient characteristics, CRRT initiation and prescription parameters, and patient outcomes (mortality, length of stay, duration of AKI) using ANOVA (for normally distributed variables) or Kruskal-Wallis (for non-normally distributed variables). Multivariate proportional hazards models will be constructed, with initial inclusion of variables with <0.2 significance in univariate analyses. Additional analyses of registry data will be developed based on specific research questions.

Data analyses will be performed by Technomics Research, LLC (Minneapolis, MN) and guided by the CRRTnet registry board.

## Discussion

Severe AKI remains a common complication of critically ill patients, and recent studies suggest that the incidence of dialysis-requiring AKI is increasing [18]. CRRT has emerged as the recommended dialysis modality for hemodynamically unstable patients in the ICU, and a recent study demonstrates that CRRT is the most commonly used dialysis modality in critically ill patients with AKI [19]. CRRT provides the advantages of improved solute and fluid balance control compared to patients on intermittent therapies. Yet in reality “CRRT” is an umbrella term that encompasses multiple different modalities and approaches. A high degree of variation exists in how CRRT is prescribed and delivered between (and within) medical centers, and there is currently a paucity of high-quality evidence to guide best practices. CRRTnet was developed with the goal of establishing the largest registry of CRRT patients to date, allowing for comparison of outcomes between a variety of different CRRT practices.

Ideally, each aspect of therapy would be assessed in randomized clinical trials. However, given the complexities of critically ill patients and potential difficulties with recruitment in this population, such an approach may not be feasible to assess every aspect of CRRT care. As such, observational studies remain an important tool, particularly when significant variations in practice naturally exist. As an example, CRRTnet was modeled after the Prospective Pediatric CRRT registry (ppCRRT) [20]. Over a 4 year period and including 13 centers in the United States, the ppCRRT enrolled 370 critically ill children who underwent CRRT and characterized outcomes in this previously under-described population [21]. Subsequent studies from ppCRRT explored patient outcomes related to vascular access, [22] anticoagulation, [23] and nutritional practices [24]. These studies provided important insight into CRRT practices and outcomes in children, spurred additional research, and continue to guide best practices in this population by informing the KDIGO AKI guidelines. Although there is significantly more literature regarding CRRT practices in adults, most of this literature is comprised of relatively small studies, primarily from single-centers. As such, there remains a significant need for large, multi-center studies such as CRRTnet.

Beyond descriptive analyses of patient characteristics and overall outcomes, we are planning on several comparative analyses. One particular area of interest is in the management of fluid overload. Our data will allow determination of fluid overload status at time of CRRT initiation, as well as daily fluid balances thereafter. While the dangers of established fluid overload have been increasing recognized in recent years, [10, 25, 26] there is a paucity of data examining different approaches to fluid management utilizing renal replacement therapy. We will examine rates of fluid removal (e.g. in ml/kg/h) and fluid overload correction to determine potential impact on patient outcomes such as renal function recovery and mortality. We hypothesize that, after adjusting for severity of illness, higher fluid removal rates will be associated with lower likelihood of renal recovery.

Another area of particular interest is anticoagulation to maintain CRRT filter patency. The KDIGO guidelines suggest regional citrate anticoagulation as the first-line method in patients without a contraindication. We anticipate that citrate anticoagulation will be associated with longer circuit life compared to heparin or no anticoagulation, a finding that has been observed in a number of clinical trials [27–30]. However, there is no consensus approach to citrate anticoagulation; while several different protocols have been published, there have been no comparative trials. Because of existing differences in citrate anticoagulation protocols among CRRTnet centers, we will be able to compare outcomes between different protocols.

One novel aspect of CRRTnet is the capture and analysis of machine data. These data record all machine measurements to the second, and include pressure measurements (e.g. access and return line pressures, filter pressures, transmembrane pressures), alarms and response to alarms, any changes in prescription (including blood, dialysate and replacement flow rates), interruptions, and actual delivered therapy. To our knowledge, this is a relatively untapped source of information, and one goal of CRRTnet will be to develop algorithms for processing the information downloaded from the CRRT machine memory cards. One of the challenges of CRRT is the complexity of therapy and the increased associated workload compared to intermittent therapies [31]. By utilizing the detailed treatment data, we will be able to characterize alarm frequency and provide more precise estimates on interface time required to maintain this therapy. This information may be helpful to administrators in determining nursing models and CRRT educational programs. In addition, nursing workload can be considered an important secondary outcome that may vary between and within centers depending on variations in CRRT protocol. For example, we hypothesize that regional citrate anticoagulation will be associated with less workload compared to non-citrate anticoagulation. It will also be interesting to compare associated workload between different citrate protocols, especially if circuit filter life is similar.

An important strength of our study is that we are examining everyday practices. While findings from controlled clinical trials can sometimes be difficult to duplicate in the “real world” setting, we will provide a relatively unfiltered look. This may be particularly important when examining adverse events and complications of therapy. Conversely, it is important to recognize that CRRT is performed at a relatively high frequency at each of the study sites, which facilitates development of local expertise and maintenance of competency. Whether or not similar practices will result in similar outcomes at smaller centers with lower volumes is uncertain. Nonetheless, we believe that CRRTnet will provide important outcomes benchmarks for centers performing CRRT.

Another important strength of CRRTnet is the ability, through waiver of consent, to include all eligible participants. In terms of the practicality of obtaining informed consent for an observational study in a population similar to our proposal, much can be learned from the unfortunate experience of the PICARD study investigators where patients and substitute decision makers were approached for informed consent [32]. Enrollment was poor, with only 52% of eligible patients included, greatly hindering the generalizability of the results. Patient refusal was infrequent at all sites, suggesting that patients themselves did not have concerns or objections to having their data collected when they were able to be approached directly. However, refusal by family occurred in almost one fifth of instances, and the absence of family or appropriate proxy was listed as a cause for refusing enrollment in as high as 40% at one site. Finally, many patients could not be enrolled because of death or discharge before being seen by study personnel. Thus, countless resources were spent on conducting an observational trial that missed almost half the eligible subjects, whose data very likely would have influenced the results of the study were they included. The CRRTnet database will be highly generalizable based on the inclusion of most eligible patients.

Some limitations of our study design are worth noting. First, we will truncate CRRT data at 14 days, which will limit applicability in patients on prolonged courses of CRRT. This decision was made primarily as a feasibility measure to limit the workload associated with detailed clinical data collection; importantly, preliminary data from participating centers showed that median duration of CRRT was in the range of 6–8 days, so we will still capture the full CRRT course for the majority of patients enrolled. Second, we will not be enrolling patients with AKI who only receive intermittent dialysis therapies and we recognize that there can be significant variation in which patients are chosen to undergo CRRT versus intermittent therapies. However, it is important to note that our goal is not to establish the role of CRRT in AKI management, but rather to focus on practice variation within CRRT prescription and delivery. So while we will not be able to advocate for which patients should be managed with CRRT, we hope to inform clinicians of best practices once a decision to initiate CRRT is made.

Over the past 30 years, CRRT has developed into an important tool for the management of critically ill patients with renal failure. Despite overall improvements, there remains marked variation in CRRT practices which is driven in part by lack of evidence-based guidance. The primary goal of CRRTnet is to develop a large data repository of CRRT patients with detailed information regarding specific CRRT practices which can then be compared in relation to outcomes. We anticipate that CRRTnet will provide the nephrology and critical care communities with an important resource for planning future clinical trials as well as for benchmarking clinical outcomes.

## Trial status

Recruitment is currently active at the 4 medical centers mentioned above. A fifth center is undergoing evaluation to join CRRTnet. The initial planned recruitment phase is 5 years with a target enrollment of 2000 patients.

## Abbreviations

AKI: Acute kidney injury
CRRT: Continuous renal replacement therapy
ICU: intensive care unit
IHD: Intermittent hemodialysis
KDIGO: Kidney Disease: Improving Global Outcomes
SCr: Serum creatinine
